# Systematic review of innate immune responses against *Mycobacterium tuberculosis* complex infection in animal models

**DOI:** 10.3389/fimmu.2024.1467016

**Published:** 2025-01-30

**Authors:** Luisa Maria Nieto Ramirez, Carolina Mehaffy, Karen Marie Dobos

**Affiliations:** Department of Microbiology, Immunology and Pathology, Colorado State University, Fort Collins, CO, United States

**Keywords:** early immunity, preclinical models, *in vivo*, cytokines, receptors, innate cells, trained immunity

## Abstract

**Background:**

*Mycobacterium tuberculosis (Mtb)* complex (MTBC) includes ten species that affect mammals and pose a significant global health concern. Upon infection, *Mtb* induces various stages in the host, including early bacterial elimination, which may or may not involve memory responses. Deciphering the role of innate immune responses during MTBC infection is crucial for understanding disease progression or protection. Over the past decade, there has been growing interest in the innate immune response to *Mtb*, with new preclinical models emerging.

**Methods:**

We conducted a systematic review following PRISMA guidelines, focused on innate immune mediators linked to protection or disease progression in animal models of MTBC infection. We searched two databases: National Library of Medicine and Web of Science. Two researchers independently extracted data based on specific inclusion and exclusion criteria.

**Results:**

Eighty-three articles were reviewed. Results were categorized in four groups: MTBC species, animal models, soluble factors and innate pathways, and other molecules (metabolites and drugs). *Mtb* and *M. bovis* were the only species studied. P2X7R receptor's role in disease progression and higher macrophage recruitment were observed differentially after infection with hypervirulent *Mtb* strains. Mice and non-human primates (NHPs) were the most used mammals, with emerging models like *Galleria mellonella* and planarians also studied. NHPs provided insights into age-dependent immunity and markers for active tuberculosis (ATB). Key innate immune factors/pathways identified included TNF-α, neutrophil recruitment, ROS/RNS responses, autophagy, inflammasomes, and antimicrobial peptides, with homologous proteins identified in insects. Metabolites like vitamin B5 and prostaglandin E2 were associated with protection. Immunomodulatory drugs targeting autophagy and other mechanisms were studied, exhibiting their potential as therapeutic alternatives.

**Conclusion:**

Simpler, physiologically relevant, and ethically sound models, such as *G. mellonella*, are needed for studying innate responses in MTBC infection. While insects lack adaptive immunity, they could provide insights into “pure” innate immune responses. The dissection of “pure,” “sustained” (later than 7 days post-infection), and trained innate immunity presents additional challenges that require high-resolution temporospatial analytical methods. Identifying early innate immune mediators and targetable pathways in the blood and affected tissues could identify biomarkers for immunization efficiency, disease progression, and potential synergistic therapies for ATB.

## 1 Introduction

In recent decades, there has been an increasing interest in the innate response mediators against members of the *Mycobacterium tuberculosis (Mtb)* complex (MTBC). MTBC groups ten genetically related species of the *Mycobacterium* genus that cause tuberculosis (TB) in different mammal species. Within MTBC, *Mtb* and *M. bovis* are the most significant species for human health to date (1, 2). *Mtb* is the leading infectious killer for humans, which generated an estimated close to 1.3 million deaths worldwide in 2023 (3). On the other hand, *M. bovis* is the primary causative agent of bovine TB and represents a risk for humans and other mammal species due to its ability to infect a broad spectrum of hosts (4, 5).

Innate immunity is the body’s first line of defense against pathogens, present from birth, and characterized by non-specific responses that do not involve genetic rearrangement (6). It aims to control infections either directly through effector responses or by activating adaptive immunity (7, 8). While much of our knowledge about innate immune responses to MTBC comes from *in vitro* and *ex vivo* studies, some findings have been inconsistent, for instance the role of neutrophils and the vitamin D during *Mtb* infection (9–11). Neutrophils’ ability to kill *Mtb* varies, depending on the cell of origin (mouse vs. human) or other unidentified factors (11). Besides these inconsistencies, these short-lived cells present significant challenges during *in vitro* or *ex vivo* assays (11–13). Similarly, although vitamin D inhibits *Mtb* growth *in vitro*, its clinical use in TB treatment has not shown substantial improvement in patient outcomes (9). These discrepancies highlight the difficulties in translating *in vitro* findings to clinical settings.

Early innate responses to *Mtb* are critical in determining the infection’s outcome in humans, with only 20-25% of individuals exhibiting signs of infection after being exposed to this pathogenic bacterium (14). In some individuals, a combination of innate and adaptive immunity, or in others, the action of mostly (or solely) innate immunity are proposed to control the infection (15–18). For instance, close contacts of TB patients (exposed to the bacterium) never develop disease or exhibit delayed hypersensitive type IV response (PPD-negative). These “self-controlling” TB cases and non-infected contacts drive the hypothesis that some *Mtb*-infected people develop innate responses with a sterilizing activity against the bacterium (19). This hypothesis has yet to be fully confirmed since recent studies have not found a protective innate response among PPD-negative contacts. However, this still does not exclude the possibility of unknown innate markers associated with early *Mtb* elimination and even “innate memory” responses that help in this matter (20, 21). While adaptive responses (led by T-cell immunity) are well-studied, they do not fully explain protection against TB, highlighting the importance of further research into innate immunity to find markers of protection or disease control.

Various preclinical animal models, including mice, non-human primates (NHP), and other vertebrates, have been used to study *Mtb*-host interactions. However, ethical concerns around animal research as well as the high cost and infrastructure associated, have led to increasing interest in alternative models, such as invertebrates. These invertebrate models hold potential for providing new insights into the *Mtb*-host interaction and expanding research options while acknowledging ethical concerns. Considering the importance of animal models in pre-clinical studies, we conducted a systematic review of recent findings towards innate responses found exclusively during *in vivo* infection with members of the MTBC, exploring the evolution and some limitations observed.

## 2 Methods

### 2.1 Study design

This systematic review aims to comprehensively analyze published peer-reviewed articles within the last decade to determine the molecules and pathways associated with innate responses in animal models infected with MTBC. We adopted the PICO (Population: animal models, Intervention: infection with an MTBC strain in the laboratory setting; Comparation: Group of non-infected animals, Outcome: innate response molecules associated with protection or disease progression) framework (22) to formulate the central question: *What type of innate immune mediators (soluble factors and innate pathways) are commonly identified among different animal models that are either associated with protection or disease progression after* MTBC *infection?* The collection of experimental studies and the analysis of their findings were focused on experimentally infected animals only and provided the most common and recently used animal models for MTBC infection. Our review also includes the description of novel host molecules associated with an early antibacterial response and potential limitations in this field. This review aims to provide state of the art information about the preclinical models used to study innate responses against *Mtb* and other members of the MTBC complex, extracting some commonalities, advantages and disadvantages, as well as some valuable findings in the most novel models used.

### 2.2 Search strategy and selection criteria

For the systematic search, two databases were consulted: the National Library of Medicine National Institutes of Health (PUBMED) and the Web of Science (WOS). The Medical Subject Headings (MeSH) terms selected were “Innate Immune Response,” “Mycobacterium tuberculosis,” and “Animal Model,” separated by the Boolean operator AND. The initial search was conducted on July 18, 2023, and the inclusion criteria were articles that:

1. were published in the last ten years,
2. described experimental data on animals,
3. reported animals infected with members of MTBC complex, and
4. evaluated innate responses.

We excluded articles focused only on *in vitro, ex vivo*, clinical studies, other infections (including other species of the *Mycobacterium* genera), studies that did not use live bacteria to infect the animals, reviews, opinions, meeting reports, and perspectives.

### 2.3 Study selection and data extraction

Two researchers (LMNR and CM) conducted the article search independently using the MeSH terms described above and extracted the articles obtained using Zotero and Endnote, respectively, following the referred Reporting Items for Systematic Reviews and Meta-Analyses (PRISMA) guidelines (23). The search was evaluated for duplicate entries, and every abstract was then analyzed for an initial application of the inclusion/exclusion criteria listed in the previous section. For most cases, the whole article was assessed in the initial screening, looking for the innate immune response findings derived from animal infections only. The total list was revised twice to manually extract all the information used in the qualitative and frequency analysis of four different categories: (i) MTBC species, (ii) animals used, (iii) soluble and membrane-associated factors and innate pathways, and (iv) other relevant molecules.

## 3 Results

### 3.1 Results of the search

**Figure 1:**
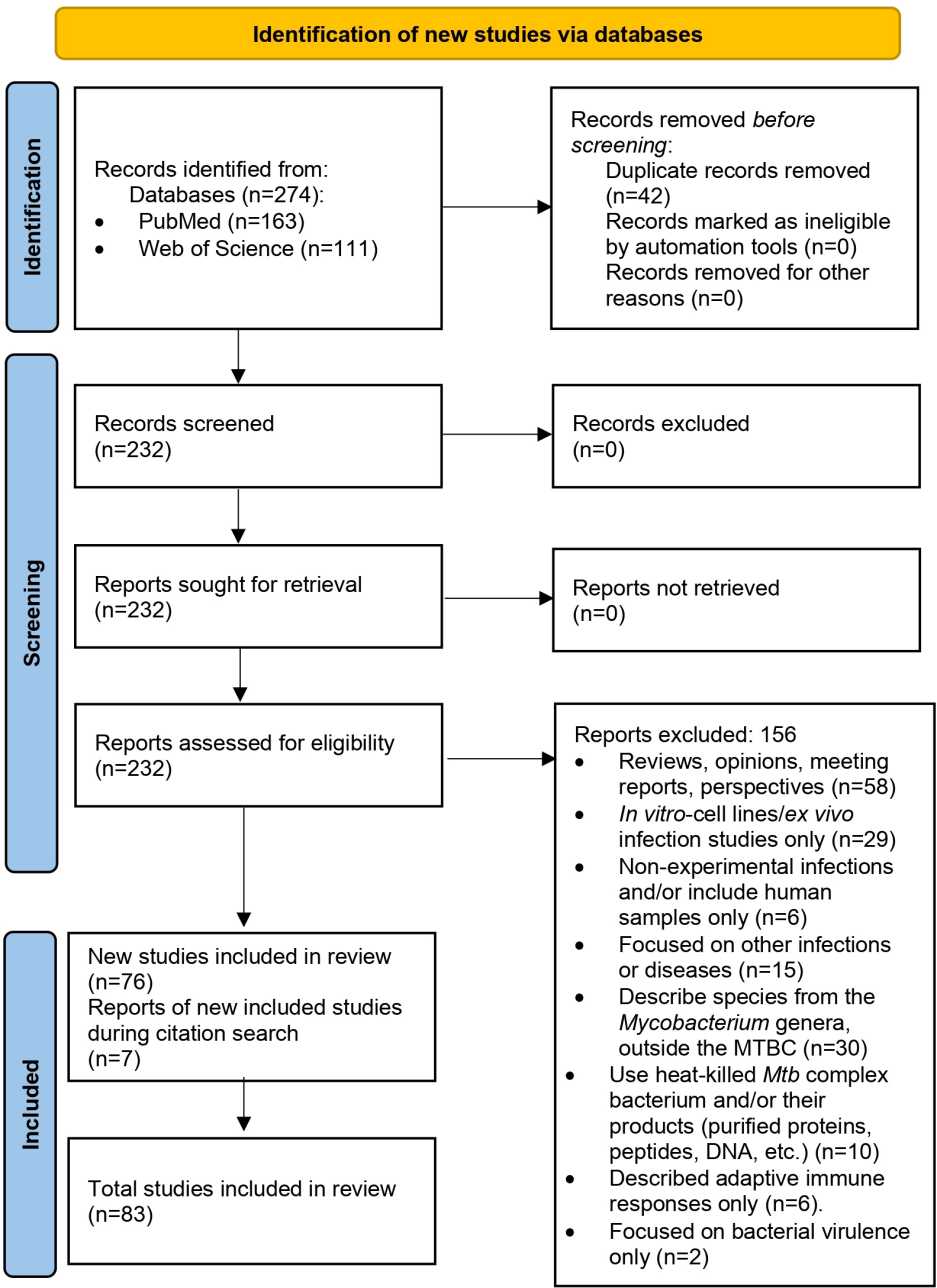
PRISMA 2020 flow diagram used in this systematic review (23).

After removing duplicates and applying inclusion and exclusion criteria, we initially selected 76 articles for the review, out of 274 articles collected for analysis. While reviewing the selected articles, we found seven additional publications that meet the inclusion criteria (some were part of the references in the initial selection), giving us 83 articles to review (**Figure 1**; **Supplementary Table 1**).

### 3.2 Synthesis and analysis of the reviewed articles

**Figure 2:**
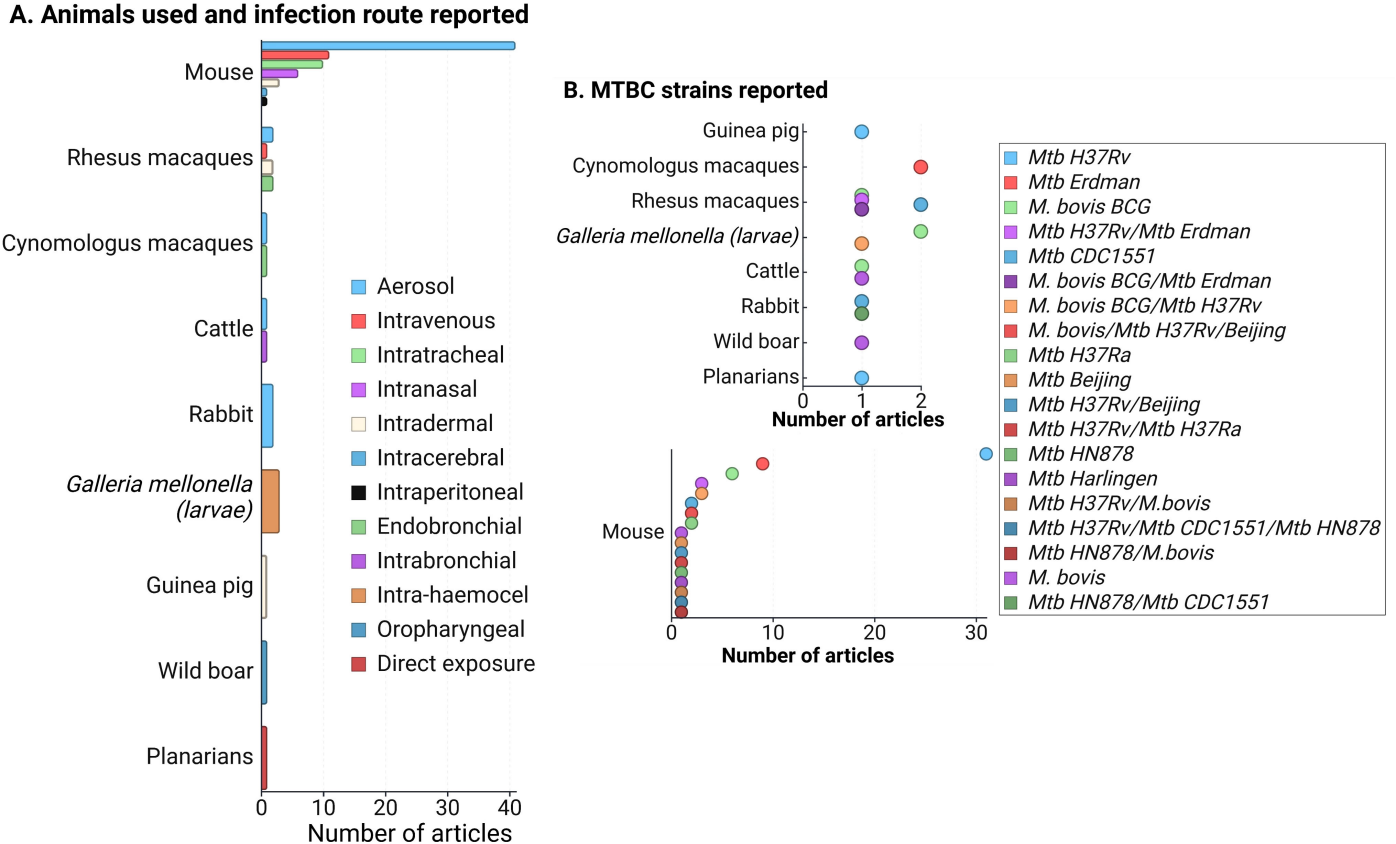
**(A)** Frequency of animals used in innate response studies against members of the *Mycobacterium tuberculosis* complex (MTBC), along with the infection routes reported for each animal model. **(B)** MTBC strains used in the reviewed articles, categorized by animal models. Since mice were the most commonly reported model, the lower section of the figure focuses specifically on MTBC strains used in mice. A total of 83 articles were evaluated. Note that some articles utilized more than one animal model (primarily mice and non-human primates), infection route, or strain, but no studies involving mixed infections were included. Figure created with Biorender.

**Figure 3:**
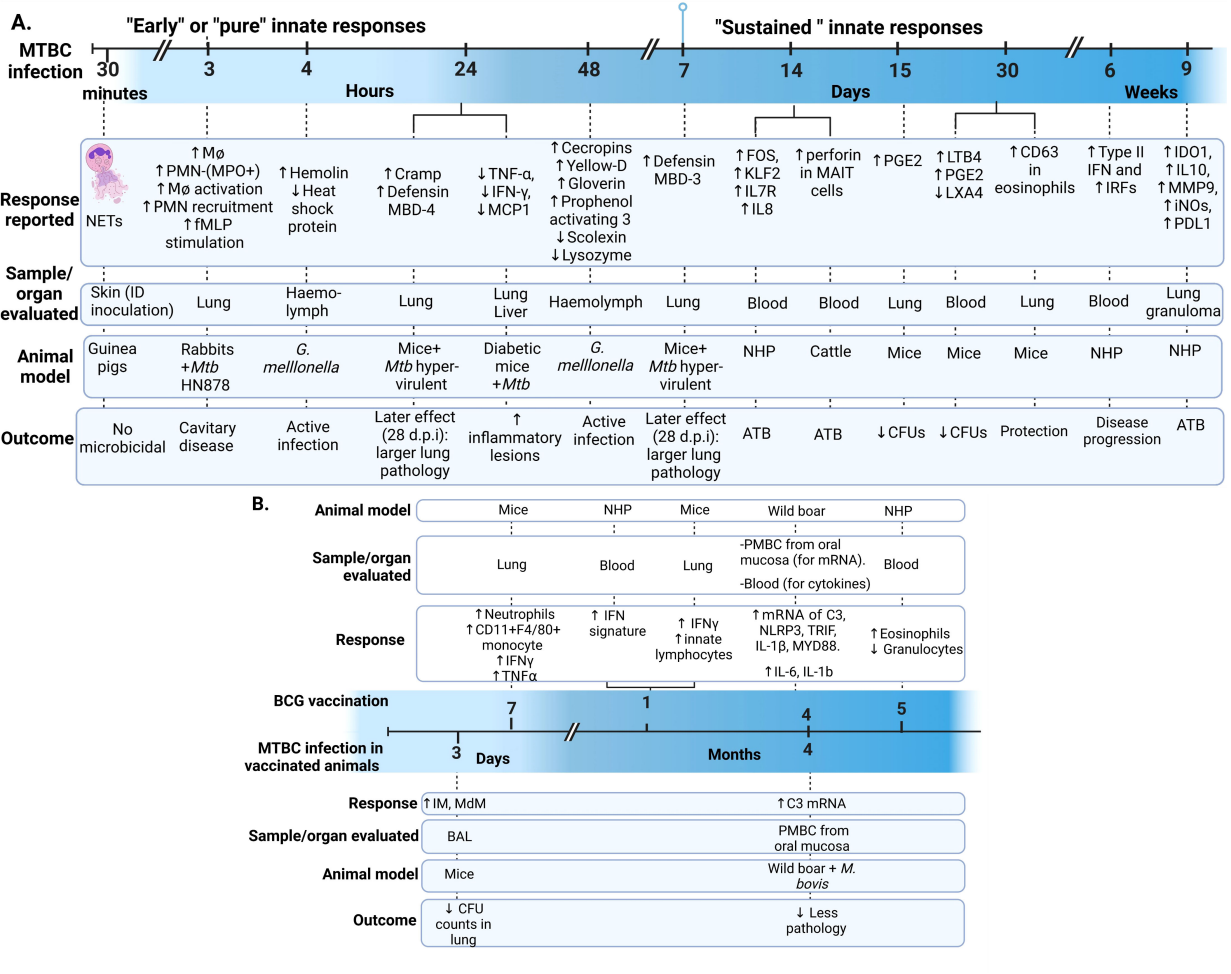
Early and sustained innate responses found in animals with active TB. **(A)** Main responses associated with active disease. **(B)** Responses observed after immunization and after *Mycobacterium tuberculosis* complex (MTBC) infection in immunized animals (29–33). Neutrophil extracellular traps (NETs). ID, intradermal; PMN, Polymorphonuclear leukocyte; fMLP, N-formyl-Methionyl-Leucyl-Phenylalanine; MPO, Myeloperoxidase; Cramp, Cathelicidin-related antimicrobial peptide; ATB, active TB; TNF, Tumoral necrosis factor; IFN, Interferon; MCP-1, monocyte chemotactic protein-1; FOS, Finkel-Biskis-Jinkins osteosarcoma, member of the AP-1 (activator protein-1) family of inducible transcription factors; KLF2, Kruppel-like factor 2; IL, Interleukin; NHP, Non-human primate; MAIT, mucosal-associated invariant T; (PGE2, Prostaglandin E2; LTB4, Leukotriene B4; LXA4, Lipoxin A4; IRF, Interferon regulatory factors; IDO1, indoleamine 2,3-dioxygenase; MMP-9, matrix metallopeptidase 9; iNOs, inducible nitric oxide synthase; PD-L1, programmed death-ligand 1; IM, interstitial macrophages; MdM, monocyte-derived macrophages; BAL, bronchoalveolar lavage; PBMC, Peripheral Blood Mononuclear Cells; C3, Complement 3 protein; NLP3, “NOD-like” receptor (NLR) pyrin domain-containing protein 3; TRIF, Toll/IL-1R domain-containing adaptor-inducing IFN-β; MYD88, myeloid differentiation primary-response 88 protein; d.p.i, days postinfection.

During the data extraction process, we focused specifically on innate immune responses observed *in vivo* following infection with live bacteria, excluding studies that used mixed approaches (like infecting animals and then evaluating responses only after *ex-vivo* stimulation of specific cell types). As an initial step, we identify the infecting bacteria, the animals and infection routes used to ensure the validity of the findings in the context of our PICO question (**Figure 2**). This review concentrates on early innate responses, as defined by innate immunity, but also included studies that measured responses weeks or even years after infection, particularly for investigating memory-like or trained immunity responses (24, 25). Long-term innate responses have been described in chronic aseptic and septic conditions, including atherosclerosis and HIV respectively (26–28). Therefore, it is not surprising to see active innate-associated pathways persisting overtime in a chronic infection like TB. We differentiated major “early” and “sustained” innate immunity responses, using the seven days break point [prior to induction of adaptive immunity (29)] to differentiate these two responses (**Figure 3A**). In **Figure 3B**, we also separated those innate responses specifically induced after BCG vaccination and after infection in the vaccinated animals.

Trained immunity occurs when pathogens or their components [pathogen-associated molecular patterns (PAMPs) and damage-associated molecular patterns (DAMPs)] induce epigenetic changes, such as histone methylation, leading to non-permanent changes in inflammatory gene expression (34). This results in a more robust immune response upon subsequent infection (24, 30, 31, 34). These epigenetic changes associated with memory responses have been reported in monocytes and hematopoietic stem cells (HSC) after BCG vaccination, leading to increased anti-mycobacterial responses (29, 35). Interestingly, virulent *Mtb* strains can suppress this process by reprogramming HSCs via type I interferon (IFN), in contrast to the type II IFN pathway induced by BCG, which demonstrates that trained immunity also depends on the infecting bacterial species or strain (36). We included four articles that explicitly described trained immunity in mice and calves (29–32), Other articles reported the effects of BCG protection in wild boars (37) or their potential in trained immunity in non-human primates (NHP) (33).

The present review does not intend to provide a hierarchy classification of the animal models studied based on which could be more relevant to replicating human ATB. Rather, it intends to provide major applications and limitations observed within each study type. Also, following our PICO question, we evaluate the main findings in terms of molecules and innate pathways, as well as their association with the clinical presentation of the disease.

#### 3.2.1 MTBC species

The reviewed articles focused on two species from the MTBC: *Mtb* and *M. bovis*, including various genotypes, attenuated or hypervirulent mutants, and clinical isolates (**Figure 2B**). Additional searches for other MTBC members, such as *M. africanum*, *M. canetti*, *M. caprae*, *M. microti*, *M. mungi*, *M. orygis*, *M. pinnipedii*, and *M. suricattae*, did not yield any result. *Mtb* H37Rv, mostly used to infect mice, was the most frequent strain reported, followed by *Mtb* Erdman and M. *bovis* BCG. Thirty articles were excluded because they reported infections with species outside the MTBC, primarily *M. marinum*. While *M. marinum* is often used to study host-pathogen interactions in zebrafish (which develop granuloma-like structures similar to those in human TB) (38), it is not a member of the MTBC and, therefore, did not meet the inclusion criteria for this review.

Different *Mtb* strains, such as H37Rv, CDC1551, and W-Beijing, were evaluated for their role in infections. Hypervirulent strains like W-Beijing, for instance, are known to cause severe pathology, including significant neutrophil infiltration and higher levels of necrosis (39). Amaral et al. showed that mice lacking the purinergic P2X7 receptor (P2X7R), which detects extracellular ATP, experience reduced myeloid cell infiltration in the lungs and less severe disease when infected with W-Beijing or the pathogenic *M. bovis* strain MP287/03, but not so much with H37Rv. This suggests that the innate receptor P2X7R plays a key role in contributing to the clinical presentation of the more aggressive forms of TB (**Table 1**) (47). Additionally, rabbits infected with different *Mtb* strains (Beijing HN878 or CDC1551) exhibited strain-specific patterns of interleukin (IL)-17 (additional information can be found in the section 3.2.3.11) (55).

**Table 1:** Examples of genetically modified mouse strains used for the evaluation of innate responses against *Mycobacterium tuberculosis* complex (MTBC)*.

Mutant Mouse strain	Factor studied	Major findings in the mutant mouse strain	Role in MTBC infection
*Cish* (-/-) (40)	Cytokine-inducible SH2-containing protein (CISH2)	Increased bacterial burden in lungs and spleen in the first weeks of infection, with reduced levels of iNOs and TNFα, independent of neutrophil levels.	Protection
*clecsf8* (-/-) (41)	C-type lectin receptor (CLECSF8)	Increased lung inflammation, bacterial counts at 4 m.p.i, and mortality. CLECSF8 recognizes TDM and is anti-mycobacterial in mice. Significant increase of neutrophil infiltration in lungs 48 h.p.i with BCG, H37Rv, or Beijing. All proinflammatory cytokines evaluated and IL-10 were significantly increased after infection with H37Rv, but only TNF-α, IFN-γ, and G-CSF after BCG infection.	Protection
TNF (-/-) (42)	Tumor necrosis factor (TNF) (during intracerebral infection)	Reduced antigen presentation capacity (↓MHCII CD80 and CD86 markers at 3 w.p.i) in macrophages and DCs, increased neutrophils and phagocytic cells recruitment with an unregulated inflammatory response and bacteria replication that resulted in higher mortality.	Protection
NsTNF (-/-) (42)	Neuron-specific TNF (during intracerebral infection)	Resistant to infection as the wild type, and therefore NsTNF was considered functionally redundant to TNF.	No effect observed
TCRβ (-/-) (43)	T cell receptor β (TCRβ)**	Increased expression of cell-surface activation markers (↑MHCII, CD86, CD80, and CD40), cytokines (↑TNF-α, IL-12, IL-6), chemokines (↑CXCL8, 9 and 11, CCL5, and CCR2), and ↑MMP1; overall associated with tissue damage.	Protection
Park2 (-/-) (43)	E3 Ubiquitin Protein Ligase (Parkin)	Unable to control intracellular bacteria and rapidly succumb after infection (at 85 d.p.i). Parkin is essential to early control of intracellular mycobacteria in lungs and other organs.	Protection
Cramp (-/-) (44)	Cathelicidin-related antimicrobial peptide (Cramp)	More severe lung and spleen lesions, and higher bacterial burden at 56 d.p.i. KO animals succumbed earlier to *Mtb* infection (at 69 d.p.i).	Protection
bhlhe40 (-/-) (45)	Transcription factor basic helix-loop-helix family member e40 (bhle40)	Higher bacterial loads in the lung, neutrophil-dominated inflammation (since 21 d.p.i), and early mortality (at 40 d.p.i) after *Mtb* infection; in a phenotype dependent on IL-10 levels. Blhe40 controls the IL-10 expression.	Protection
IL-21R (-/-) (46)	IL- 21 Receptor	IL-21 acts as a potent inhibitor of an IL-17A-producing γδ T-cell subset that mediates neutrophil-dependent inflammatory responses during BCG infection.	Not evaluated
P2X7R (-/-) (47)	P2X purinoceptor 7	Disease attenuation with moderate lung pathology, leukocytes infiltration (on 28 d.p.i) and delayed in mortality in an ATP-dependent manner after infection with the hypervirulent Beijing *Mtb* strain only.	Disease progression (with hypervirulent strains).
DuoX1 (-/-) (48)	Dual oxidase 1 (Duox1)	Increased the pro-inflammatory cytokine in the airway at day 30 post-*Mtb* infection, without differences in bacterial counts or lung pathology.	No effect observed
fcgrt (-/-) (49)	Neonatal Fc Receptor (FcRn)	Increased levels of CD103^+^ DCs population pre-infection and at 14 d.p.i, with a later decrease of neutrophils, monocyte and macrophages at one m.p.i in the granulomas. This effect was concomitant to transient reduction in CFU counts.	No effect observed
Mif−/− (50)	Macrophage migration inhibitory factor (MIF)	Decreased IL-6, TNF-α, IL-10, and IL-12 and increased IFN-γ, G-CSF, MIP-2 in lungs at 2 m.p.i. Larger lesions, higher bacterial counts and greater accumulation of neutrophils in the lung with high MPO^+^, resulting in faster mortality compared to WT after infection.	Protection
NOD/SCID/IL-2Rγ-/- (NSG) (51)	Human engrafted macrophages in NSG mice	Human macrophages in severely immunodeficient mice support BCG growth better than murine macrophages from C57BL/6 mice at 4 w.p.i.	Higher susceptibility
B6 ΔdblGata and B6 PHIL (eosinophil-deficient) (52)	Eosinophils	Downregulation of genes involved in short-chain fatty acid, endocannabinoid, and arachidonic acid metabolism and neurological processes at 90 d.p.i. compared to WT. KO animal exhibited higher bacterial loads after 30 d.p.i. and died earlier. No changes observed in lung-resident immune cells or pathology.	Protection
*Atg5fl/fl-Lysm-cre* (53)	Autophagy related gene 5 (ATG5)	Increased bacterial load in lung, severe lung inflammation with large lesions since 3 w.p.i. Mutant animals died at 40 d.p.i. ATG5 in contrast to other ATG genes is essential to control *Mtb* infection.	Protection
*Ifnar*(-/-) (54)	Type I IFN receptor and αGM-CSF	Blockage of GM-CSF causes excessive formation of NETs, increased CFU counts and lung pathology at 21 d.p.i in WT. This process was dependent on Type I IFN responses, since this trend was mitigated in the KO animal. Type I IFN induced neutrophil response was linked to disease exacerbation.	Higher susceptibility

*Evaluating innate responses measured after *in vivo* infection only, excluding any ex-vivo post stimulation findings.

**Although this receptor is part of adaptive immunity, innate responses were also evaluated in this knock out (KO) strain and summarized here. iNOs, inducible nitric oxide synthase; TNF, tumoral necrosis factor; TDM, trehalose dimycocerosate; BCG, Bacillus Calmette-Guérin; IL-, interleukin; IFN, interferon; G-CSF, Granulocyte colony-stimulating factor; MHC, Major Histocompatibility Complex; DCs, dendritic cells; w.p.i, weeks post-infection; CXCL, Chemokine (C-X-C motif) ligand; CCL, Chemokine (C-C motif) ligand; CCR2, C-C chemokine receptor type 2; MMP, matrix metallopeptidase; d.p.i, days post infection; m.p.i, months post infection; MIP-2, Macrophage inflammatory protein 2; MPO, Myeloperoxidase; GM-CSF, Granulocyte-monocyte colony-stimulating factor; NOD, non-obese diabetic; SCID, severe combined immune deficiency; B6 ΔdblGata, B6 mice with a deletion of a high-affinity GATA–binding site in the GATA-1 promoter; B6 PHIL, Transgenic mice that express cytocidal diphtheria toxin A under the eosinophil-specific EPX promoter; NETs, neutrophil extracellular traps; WT, wild type.

Strain-specific responses were also observed in BCG-vaccinated C3Heb/FeJ mice subsequently infected with the *Mtb* Beijing strain. Henao-Tamayo et al. found a significant reduction in granulocytic influx to the lungs 25-50 days post-infection, which was accompanied by lower bacterial counts and less lung necrosis at the same time points (56).

#### 3.2.2 Animals used and significant findings in each model

Animal models used in the 83 evaluated articles are summarized in **Figure 2A**, highlighting mammalian species as the most frequently used. Similarly to previous reviews focused on animal models of *MTBC* infection (57–60), most of our findings are dominated by the responses observed in mice and NHP. The aerosol route was highly reported among infected mammals (except for the wild boar that was infected via the oropharyngeal route only) (**Figure 2A**), which is related to the need to use animal models that better replicate the infection route for human infection, especially for the case of *Mtb.* The infection route was a relevant variable (especially for mammals), since that could also determine some of the evaluated tissues and the type of host response expected.

##### 3.2.2.1 Mouse

Many important discoveries in the innate response against *Mtb* (i.e., role of TNF-α, IFN-γ, IL-12, among others) have been obtained in the mouse model (61). Mice have also been used to study the most recently described role of micro-RNAs (like miR-223 and miR-155) (62, 63) and sensing pathways for bacterial nucleic acids in MTBC (64). Similarly, innate receptors such as TLR2, TLR9, the complement system, and other conserved immunological pathways in mammalians have been studied in the mouse model. Although they do not entirely mimic the human TB infection, mice provide similar infection routes to humans, cost-effective use of several biological replicates, and the availability to explore different genetic and immunological tools (65). Recent studies have shown that lower bacterial doses in mice, like the ultra-low dose (1-3 founding bacteria, measured using DNA-barcoded strains) can more successfully mimic human infection (61, 66).

Under the mouse background (BALB/c, C57BL/6, and C3HeB/FeJ, also known as Kramnik), transgenic and knock-out strains for specific genes have been used as models to evaluate different innate immune response mediators in the articles reviewed (**Table 1**). These different genetic mice also reflect different degrees of susceptibility to *Mtb* infection. C57BL/6 mice are generally considered more resistant to *Mtb* infection than BALB/c mice, and both mice do not develop necrotic lung lesions, a pathology hallmark of human TB (67). On the contrary, C3HeB/FeJ mice are highly susceptible and fail in controlling *Mtb* infection, displaying a more severe disease progression with characteristics closer to human TB, including the development of large, caseous lung necrotic lesions (68–70).

###### 3.2.2.1.1 Study of innate immune responses in the context of metabolic comorbidities

The mouse model was used to evaluate chronic comorbidities, specifically type 2 diabetes (T2D), which negatively impacts *Mtb* infection outcomes in humans (51, 71, 72). In this review, we found an overall similar cytokine profile in lung and liver from diabetic mice infected with *Mtb* and *M. bovis* intravenously, with earlier and stronger responses after *Mtb* infection (**Figure 3A**, **Table 2**) (71, 72). A systemic increase in pro-inflammatory cytokines characterizes T2D (107). The results observed in this review highlight the bi-directional relationship between T2D and susceptibility to TB, associated with a dysregulated cytokine profile (71, 72). The similar cytokine trend reported post-*Mtb* and *M. bovis* infection in this mouse model does not exclude additional innate responses differentially induced by these two species that could be explored deeper, for instance using a discovery mass spectrometry approach (108, 109).

**Table 2:** Molecules associated with main innate pathways reviewed during *Mycobacterium tuberculosis complex* (MTBC) infection.

Molecule	General trend observed regarding MTBC infection	Animal (s)	References
Tumoral necrosis factor alpha (TNF-α) pathway			
TNF-α	Shared trend with IFN-γ: ↓ in lung and liver of diabetic mice at 1 d.p.i with *Mtb*. ↑ expression in MAIT cells from BAL at 3 w.p.i.in NHP that develop LTBI vs ATB. ↑ after HDAC-6 inhibition by Tubastatin at 5 d.p.i with H37Ra, associated with reduced CFU counts in mice. ↓ in the intestine of microbiota disrupted mice 10 w.p.i.	Mice NHP Rabbits	(40, 43, 48, 50, 71–83)
Nf-kB	↑in S1-P treated animals that exhibit Protective immunity against *Mtb*	Mice	(84)
ROS and RNS production in phagocytes			
iNOs/NOS2	↑ in ATB. ↑ in rabbits supplemented with iron at 8 w.p.i. with no effect in bacterial burden or lung pathology. ↑by type I IFN, S1-P (in macrophages) and CISH. ↑ after Ipr1 recombinant BCG vaccination in mice and associated with protection against *Mtb*. ↓ in lungs of transgenic mice overexpressing SMAR1 since 6 w.p.i.	Mice NHP Rabbit	(40, 74, 79, 84–89)
NCF4	↑ after Ipr1 recombinant BCG vaccination and associated with protection against *Mtb*. ↓ bacterial load and pathology.	Mice	(88)
Myeloperoxidase	Present in NETs.	Guinea pig	(90)
Autophagy			
LC3	Fundamental in TB elimination and ↓disease severity	Planarians Mice	(91, 92)
ATG5	Essential to limit inflammation driven by PMN	Mice	(53, 81)
MORN2	Promote LC3-mediated autophagy	Planarians	(91)
Complement			
C3	↑ in PBMC from oral mucosa of oral-vaccinated animals with ↓ lesion scores (measured in tonsils, lymph nodes, lung) at ~4 m.p.i.	Wild boar	(37)
Sfpd	Activate the complement. ↑ after Ipr1 recombinant BCG vaccination/↓ bacterial load and pathology	Mice	(88)
Neutrophil recruitment			
MMP-9	Excessive inflammation/tissue damage. Induce NETs. ↑ after loss/blockage of GM-CSF.	NHP Mice	(54, 85)
S100a6/S100a8/S100a9/Cd17, CXCR2	↑ in lungs and blood after loss/blockage of GM-CSF. ↑bacterial growth and promotes disease severity, independent of IFN-γ.	Mice	(54)
MMP-8	Excessive inflammation/tissue damage. Induce NETs. ↑ after loss/blockage of GM-CSF.	Mice	(54)
MMP-1	Tissue damage/↑ in TCRβ−/−	Mice	(43)
Elastase	Present in NETs	Guinea pig	(90)
Prg2 (mostly found in eosinophils, but also in neutrophil degranulation)	↑ after Ipr1 recombinant BCG vaccination/↓ bacterial load and pathology	Mice	(88)
NETs containing DNA and histones	Rapid but low effect response in *Mtb* killing	Guinea pig Mice	(54, 90)
Inflammasomes			
Caspase-1	Inflammatory response and ↑in disease progression and TB pathology	NHP Mice	(93–95)
NLRP3	Part of the inflammasome complex. Cytosolic PRR sensing RD1 *Mtb* components (Esat 6). Inhibited by miR-20b. ↑Transcripts in lungs of vaccinated animals	Wild boar Mice	(37, 94, 95)
AIM2	DNA cytosolic sensor ↑ in ATB	NHP Mice	(93, 94)
Antimicrobial peptides			
LL37	cause ↓ bacterial load/pneumonia ↑pro-inflammatory response	Mice	(44, 79, 96–98)
mβD and HβD−3 and mβD−4	cause ↓ bacterial load/pneumonia ↑pro-inflammatory response	Mice	(96, 97)
Cecropin-A, A1 and D-like	↑ in BCG infected	*G. mellonella*	(99)
HAMP	Show temporal changes after iron supplementation in ATB	Rabbit	(74)
RegIII-γ (Regenerating islet-derived lectins)	↓ in small intestine of microbiota disrupted mice at 10 w.p.i.	Mice	(100)
Toll-like (TLR) receptor cascades			
TLR4	↑ antibacterial effect when activated with LPS and used in junction with rifampicin and NOD-2 ligand. ↑ in TCRβ−/− mice after *Mtb* infection. ↑ after Ipr1 recombinant BCG vaccination.	Mice	(43, 88, 101)
TLR2 and TLR9	Both can be dispensable for IL-12 production and protective responses against *Mtb*. ↑after Ipr1 recombinant BCG vaccination. ↑ in TCRβ−/− mice after *Mtb* infection. TLR2 only: ↓ in lungs from infected mice with disrupted gut microbiota at 10 w.p.i.	Mice	(43, 88, 100, 102)
Myd88	Control *Mtb* growth, restore inflammatory cytokine, signal of active innate response post-vaccination	Wild boar Mice	(37, 103)
TRAF6	↑ levels associated with TB signs and tissue damage, ↑ in TCRβ−/− mice	Mice	(43)
TRIF	Localized lung response/↑ in cases with less pathology	Wild boar	(37)
Interferon (IFN) signaling			
IRF1 IRF2 IRF4 IRF7	IRF1 ↑ in ATB IRF2 ↑ in severe TB. IRF4 ↓when ↑ TB signs IRF7 ↓in vaccinated animals and associated with disease severity	NHP Rabbits	(55, 93, 104)
IFN-γ	↑ in BAL of in mice treated with Colexib after 15 d.p.i, associated with higher phagocytic activity and reduced CFU counts. (other shared with TNF-α, see first section of the table)	Mice NHP Rabbit	(32, 50, 71–76, 83, 87, 89, 94, 105, 106)
STAT1	↑ in virulent HN978 infection	NHP Rabbit	(55, 93)
JAK2	↑ in ATB	NHP	(104)
SOCS1 and 3	↓levels associated with TB signs and tissue damage, ↓ in TCRβ−/− mice	Mice	(43)

NHP, non-human primates; IFN, Interferon; d.p.i, days post infection; MAIT, mucosal-associated invariant T; BAL, bronchoalveolar lavage; TB, tuberculosis; LTBI, latent TB infection; ATB, Active TB; HDAC, Histone deacetylase; w.p.i, weeks post-infection; S1-P, sphingolipid sphingosine-1-phosphate; ROS, Reactive oxygen species; RNS, Reactive nitrogen species; iNOs/NOS2, inducible Nitric oxide synthase/Nitric Oxide synthase 2; CISH, Cytokine-inducible SRC homology 2 (SH2) domain protein; BCG, Bacillus Calmette-Guérin; SMAR1, Scaffold/matrix attachment region binding protein 1; NCF4, Neutrophil Cytosolic Factor 4; *Ipr1*, intracellular pathogen resistance gene 1 from mouse; NETs, Neutrophil extracellular traps; LC3, Microtubule-associated protein 1A/1B-light chain 3; ATG5, Autophagy related 5; PMN, polymorphonuclear leukocytes; MORN2, Membrane Occupation and Recognition Nexus repeat-containing-2; C3, Complement C3 protein; PBMCs, Peripheral Blood Mononuclear Cell; m.p.i, months postinfection; Sfpd, Surfactant protein D; MMP, Matrix metalloproteinases; GM-CSF, Granulocyte-monocyte colony-stimulating factor; S100a6, S100 calcium-binding protein A6; S100a8 (also known as MRP8) and S100a9, S100 calcium-binding protein A8 and A9 respectively, together form a complex called calprotectin; CXCR, chemokine receptor CXC; TCR, T-cell receptor; Prg2, Proteoglycan 2; NLRP3, NOD-like receptor (NLR) pyrin domain-containing protein 3; AIM2, DNA cytosolic sensor “absent in melanoma 2”; LL37, cathelicidin; mβD and HβD, mouse and human beta-defensins; HAMP, hepcidin antimicrobial peptide; LPS, lipopolysaccharide; NOD, nucleotide oligomerization domain; IL, interleukin; Myd88, myeloid differentiation primary response 88; TRAF6, Tumor necrosis factor receptor-associated factor 6; TRIF, Toll/IL-1R domain-containing adaptor-inducing IFN-β; IRF, Interferon regulatory factor; STAT, signal transducer and activator of transcription; JAK, Janus kinase; SOCS, Suppressor of cytokine signaling.

###### 3.2.2.1.2 Trained immunity

Nucleotide-binding oligomerization domain-like receptor (NOD)-associated trained immunity response was studied in mice by Bricket et al. and was not associated with the early anti-mycobacterial mechanisms induced by BCG. In this study, mice were infected 7 days after receiving the BCG vaccine, detecting similar reductions in lung bacterial counts in both NOD1 and NOD2 deficient mice, as well as in wild-type (WT) mice. At 7 days post-vaccination, higher levels of circulating monocytes (CD11b+F4/80+) and neutrophils were recruited to the lungs, and these were sufficient to control the infection in live-BCG vaccinated mice. This response was accompanied by increased levels of TNF-α and IFN-γ in the lungs. However, the protective innate immune response was independent of natural killer (NK) cells and IFN-γ levels and was instead dependent on neutrophils (29).

Steigler et alfound significant increased levels of innate lymphoid cells (ILCs) secreting high levels of IFN-γ in the lungs, four weeks after intranasal BCG vaccination. The immune response found using the intranasal vaccination was stronger compared to other administration routes, such as intradermal vaccination (32). This finding is relevant for mucosa-associated trained immunity due to the ability of ILCs to induce memory responses (110). D’Agostino et al. showed a rapid increase of interstitial (IM) and monocyte-derived macrophages (MdM) in the bronchoalveolar lavage (BAL) as early as three days post-*Mtb* infection in previously immunized mice, while >90% of the cells in BAL were alveolar macrophages (AM) in unimmunized mice. These findings confirmed that airway macrophages (mostly represented by IM and MdM) were associated with protection (lower CFU counts) during early *Mtb* infection and their role in trained immunity (**Figure 3B**) (30). Importantly, none of the articles mentioned above describe any epigenetic or a specific biochemical mechanism associated with the innate-induced memory response.

Additional knock-out (KO) mouse strains allowed the study of specific mediators, either soluble molecules or receptors, to validate their roles in the innate response against MTBC in pulmonary or cerebral infection. **Table 1** summarizes the main findings for some of these KO mouse strains.

##### 3.2.2.2 Non-human primates

*Macaca mulatta* (rhesus macaques, RM) and *Macaca fascicularis* (cynomolgus macaques, CM), were the second most used models to study TB, due to their ability to closely mimic human TB and immune responses. NHPs are the preferred model to investigate TB vaccines and pathogenesis. While NHP models are highly informative, they have limitations, including high costs, specialized infrastructure, and ethical concerns (66). In our review, five articles used RM (33, 52, 73, 85, 93) and two used CM (104, 111). There are notable differences between NHP species in their response to *Mtb* infection. Although both RM and CM are highly susceptible to *Mtb*, RM are generally more vulnerable to severe TB than CM (66, 112). This increased susceptibility in RM may be linked to differences in their innate immune responses. RM display an anti-inflammatory profile in peripheral monocytes even before infection, with reduced IFN-γ secretion, whereas CM show a stronger pro-inflammatory response, particularly the release of TNF-α, during early infection (113). Most of our reviewed articles using NHP focused on cellular responses and blood transcriptomic profiles, which we differentiated by NHP species (**Table 3**).

**Table 3:** Reviewed non-human primate (NHP) innate responses after *Mycobacterium tuberculosis* complex (MTBC) infection*.

NHP species (authors)	Factor studied	Major findings	Role in MTBC Infection
RM (Bohrer et al.)	Eosinophils (52)	Heterogeneous infiltration of eosinophils, mainly located at the outer rim of the granuloma with evidence of degranulation prior cell death (significant high expression of CD63 and diffuse eosinophil peroxidase in the necrotic core). CD63 expression in the granuloma inversely correlated with bacterial burden at 7-12 w.p.i, but CD63 expression was highly variable even in granulomas from the same animal. Also, there was no correlation between eosinophil abundance and bacterial load.	Inconclusive (in this model)
RM (Sarfas et al.)	Classical monocytes (CD14^+^CD16^-^), granulocytes, eosinophils and CD16^+^CD56^+^ NK after BCG comparing neonatal (neo) vs adult animals (33)	BCG vaccination induces in neo: ↑eosinophils at ~20 weeks p.v. ↓ granulocytes from ~12 weeks p.v. ↓classical monocytes at ~20 weeks p.v. ↓monocytes and NK-producing IFN-γ and TNFα and ↑IL-2 producing NK since week 20 p.v.	Inconclusive (only responses measured after BCG vaccination)
RM (Singh et al.)	Poly-morphonuclear myeloid-derived suppressor cells (PMN-MDSCs) (85)	PMN-MDSCs expressing Ki67, IDO1, IL-10, MMP-9, iNOS, and PD-L1 were higher in the periphery of granuloma of ATB vs LTBI animals at 9 weeks p.i.	ATB, disease progression.
RM (Bucsan et al.)	GrB^+^IFN-γ^+^IL-17^+^TNF-α^+^ Mucosal-associated invariant T (MAIT) cells (73)	GrB^+^IFN-γ^+^IL-17^+^TNF-α^+^ MAIT cells were specifically higher in BAL of animals that control the *Mtb* infection at 3 w.p.i.	Associated with LTBI (controlled) rather than with ATB
RM (Hansen et al.)	IFN-response-associated genes (IRF7) and the inflammasome components AIM2 CASP1, and STAT1) (93) Neutrophil degranulation markers (MMP8, OLFM4 and CD52) (93)	Transcript blood levels of IRF7, AIM2, STAT1, and CASP1 at 28 d.p.i were correlated disease progression and were strongly reduced in the vaccinated animals, based on bacterial load and pathology score. Animals with a “protective profile” (based on bacterial load and pathology score) have higher levels of genes associated with neutrophil degranulation markers in blood immediately pre-*Mtb* challenge.	ATB and progression Protection
CM (Gideon et al.)	Gene clusters related to complement regulation, hematopoiesis, IFN responses, inflammation, and coagulation/platelet response. Triggering receptor expressed on myeloid cells (TREM)-1 signaling (111)	Transcriptomic analysis revealed increased expression in these gene clusters, especially IFN signaling, JAK-STAT pathway, and dendritic cell maturation, since day 20 p.i in ATB. TREM-1 signaling associated transcripts in blood were associated with higher inflammation in ATB at 90-180 d.p.i.	ATB Higher extent of lung inflammation
CM (Javed et al.)	FOS, Kruppel-like factor 2 (KLF2), IL7R and IL8 (104) Type II IFN signaling and other IFN response factors (IRFs) (including the genes SOCS3, JAK2, STAT1, SPI1, IRF1, IRF2, IRF4, IFNGR1, and GBP1) (104)	Upregulated in the first 2 weeks and then stayed downregulated in cases of ATB until week 6. This transcriptomic profile may be associated with increased apoptosis after 2 w.p.i. and stimulation of T cell response and an M2 phenotype. The upregulation of these genes was associated with ATB, some of them since 2-6 w.p.i compared to pre-infected stage. Additionally, Mauritian macaques were more susceptible to develop severe TB compared to the Chinese lineage. Downregulation of SOCS3, IRF4 and IFNβ1 with upregulation of IRF4 correlated with signs of disease.	Early marker of ATB (exposure to *Mtb*) Markers of disease progression.

*Evaluating innate responses measured after *in vivo* infection only, excluding any *ex-vivo* post stimulation findings. RM, Rhesus Macaques; CM, Cynomolgus Macaques; w.p.i and d.p.i, weeks post-infection and d.p.i respectively; p.v, post-vaccination; NK, natural killers; BCG, Bacillus Calmette-Guérin; IFN, interferon; TNF, tumoral necrosis factor; IL, interleukin; Ki67, cellular marker for proliferation discovered in the city of Kiel and with the cell clone 67; IDO1, indoleamine 2,3-dioxygenase; MMP-9, matrix metallopeptidase 9; iNOs, inducible nitric oxide synthase; PD-L1, programmed death-ligand 1; TB, tuberculosis; ATB, Active TB; LTBI, Latent TB infection; GrB, Granzyme B; AIM2, DNA cytosolic sensor “absent in melanoma 2”; CASP, caspase; STAT, signal transducer and activator of transcription; OLFM4, Olfactomedin 4; JAK, Janus kinase; SIV, Simian immunodeficiency virus; FOS, Finkel-Biskis-Jinkins osteosarcoma, member of the AP-1 (activator protein-1) family of inducible transcription factors; SOCS, Suppressor of cytokine signaling; SPI1, SPI-1 Proto-oncogene, transcriptional factor also known as also known as PU.1; IFNGR1, Interferon gamma receptor 1; GBP1, Guanylate-binding protein 1.Symbol ↑ stand for increased and for symbol ↓ stand for decreased.

###### 3.2.2.2.1 Innate responses in NHP with ATB vs LTBI

The NHP model is valuable for studying innate immune responses in the context of active TB (ATB) and latent TB infection (LTBI) (73, 85, 111). Singh et al. found that RM with ATB had a higher proportion of granulocytic polymorphonuclear myeloid-derived suppressor cells (PMN-MDSCs) in lung granulomas, particularly in the periphery near the lymphocyte core, compared to RM with LTBI. These PMN-MDSCs in ATB showed elevated expressions of markers associated with immunosuppression, such as, IL-10, Indoleamine 2,3-dioxygenase 1 (IDO1), Matrix Metalloproteinase (MMP-9), inducible nitric oxide synthase (iNOS), and programmed death-ligand 1(PD-L1). The researchers suggested that these cells, due to their localization within the lymphocytic regions of granulomas, could suppress anti-mycobacterial immune responses, making them a potential marker for ATB (85). However, the translation of this finding into the clinical setting as part of a diagnostic tool is still unclear. On the other hand, a rapid but non-significant increase of a specific population of mucosal-associated invariant T (MAIT) cells was more associated with LTBI than ATB in RM. These MAIT cells reported by Bucsan et al, were detected in the blood and BAL at three w.p.i (**Table 3**) (73).

ATB and LTBI outcome in CM were associated with early differences in the blood transcriptomic profile. Gideon et al. reported an increased transcriptomic blood signature in CM as early as 20-30 days d.p.i. that was linked to ATB (**Table 3**). Interestingly, LTBI animals showed an early increased IFN response at 7 d.p.i., which was reversed by 30 d.p.i. Overall, the transcriptomic profiles of ATB and LTBI in CM were similar to those observed in humans, though LTBI profiles in CM were more heterogeneous and less intense (111). Confirming the findings from Gideon et al. (111 *).*, Hansen et al., demonstrated the association between increased IFN transcripts in the blood of CM with ATB. They also revealed the protective effect of blood markers pre *Mtb*-challenge that were associated with neutrophil degranulation (**Table 3**) (93).

Javed et al. reported a temporal gene expression analysis in the blood of CM following *Mtb* infection, using a human genomic oligonucleotide microarray to identify biomarkers for ATB. This group identified gene clusters based on expression changes between pre-infected and infected animals over a 6-week period, described in **Table 3** (104). They also found that CM’s susceptibility to *Mtb* varied by animals’ origin, with Mauritian animals being more susceptible than Chinese ones. This was attributed to intrinsic differences in type II IFN gene expression among other genes (**Table 3**). Finally, the authors cautioned that differences in blood transcriptomics should be interpreted carefully, as they could reflect cell migration to the infection site or be influenced by higher cell death rates in specific cell types (104).

###### 3.2.2.2.2 Innate responses following BCG vaccination discriminated by age

Sarfas et al. evaluated the immune responses of neonatal-vaccinated (neo-BCG) RM versus adult-vaccinated (ad-BCG) RM. They assessed systemic responses from 12 weeks to 3 years post-vaccination in neonatally vaccinated animals and at 20 weeks post-vaccination (p.v) in adults (33). Time dependent differences in the frequency of specific subpopulation of circulating NK, monocytes, and eosinophils, as well as the levels of IFN-γ, TNF-α, and IL-2 produced by these cells were reported (33). These differences in immune cell populations and cytokine responses led the authors suggest that neonatal BCG vaccination may induce distinct innate-protective responses in infants, indicating also the induction of trained immunity at this age. They also recommended that future vaccine efficacy studies in non-human primates (NHPs) should include evaluations at neonatal ages (33). Unfortunately, these responses were not further evaluated after *in vivo* infection in vaccinated animals (**Table 3**).

Interestingly, eosinophils were also studied in the context of MTBC infection in RM and mice (52). Although eosinophils were not infected with *Mtb*, active and early recruitment of these cells positive for CD63 (degranulation marker) was observed in the lung tissue of both animal models. A protective role for these cells in the control of *Mtb* infection was established by Bohrer et al., in an eosinophil-deficient KO mouse model (**Table 1**), but a more heterogeneous response was observed in RM (**Table 3**).

##### 3.2.2.3 Other mammals

**Rabbits** were reported by two articles that evaluated pathological features and transcriptional differences in innate-associated genes after external stimuli (74) or in response to *Mtb* strains with different virulence profiles (55). Kolloli et al. concluded that iron supplementation induces different transcriptomic changes, depending on the evaluated tissue, without affecting bacterial burden or disease pathology. For instance, a systemic downregulation of IFNG, TNFA, and IL1B and upregulation of IL6 and SMAD6 was reported four weeks p.i (w.p.i). In the lungs, IL6 is downregulated similarly to the transcriptional regulators SMAD6 and SMAD7, while IL10, NOS2, IFNG, and TNFA are upregulated simultaneously. SMAD6 and SMAD7 are involved in the expression of the hepcidin antimicrobial peptide (HAMP) (74).

Subbian et al. found transcriptional differences in 14 genes at 3 hours post-infection with greater recruitment of macrophages and polymorphonuclear leukocytes (PMN) (with higher myeloperoxidase activity) and cavitary disease more like human active TB in the lungs of rabbits infected with HN878 compared to those infected with CDC1551. Overall, pathways involved in macrophage activation, fMLP (N-formyl-Methionyl-Leucyl-Phenylalanine)-stimulation or PMN recruitment and activation were upregulated after HN878 versus CDC1551 infection (**Figure 3A**). Some of the genes with increased expression include TNF, CXCL10, STAT1, IL1A, SPP1, CCL4, CCL2, IRF5, CD38, while reduced expression of IL4R, CAV1, TGFB2, IL18, and CD36 were observed (66). That early signature profile was relatively constant at four w.p.i and proposed to determine long-term *Mtb* infection in rabbits (55).

**Cattle** infected with either *M. bovis* BCG or AF2122/97 were used as a model to study trained immunity (31) or MAIT cells (114), respectively. In the study by Guerra-Maupome et al., calves did not exhibit differences in the number of monocytes expressing CD14+, CD11b+, or TLR-4 at four weeks post-BCG-vaccination, compared to unvaccinated animals (31) as opposed to the increased trend observed in BCG-vaccinated humans (115, 116). Peripheral Blood Mononuclear Cells (PBMC) from the aerosol vaccinated compared to the unvaccinated cattle showed increased levels of IL-6, IL-1β, and TNFα, but only after *ex-vivo* stimulation with LPS or Pam3CSK4. The latter allowed the authors to conclude that the innate immune system of calves can be “trained” and could be used as an immunomodulatory strategy (31). However, they did not explore the trained immunity effect after the animals were re-infected with *M. bovis*.

The second article by Edmans et al. revealed a MAIT cell population in the blood that showed higher perforin expression two-weeks after *M. bovis* infection without changes in the number of these cells. This high perforin expression was seen mainly in animals with TB lesions (114).

**Guinea pigs** develop necrotic core granulomas, and their macrophages exhibit surface CD1b and CD1c, making them a beneficial small animal model for replicating human TB (67). This animal has been widely studied for host-pathogen interactions in metabolic co-morbidities (such as diabetes and malnutrition) and mainly adaptive responses against MTBC (117). However, only one article focused on innate responses in the guinea pig was found during our review, specifically studying the neutrophil extracellular traps (NETs) (chromatin webs). Although this was one of the earliest responses observed, it was not microbicidal (90).

**Wild boars** were orally vaccinated with a heat-inactivated *M. bovis* strain and then infected with the same (viable) strain. Beltrán-Beck et al. evaluated the transcriptomic responses post-vaccination in the PMN of the oral mucosa and serum and then after infection in vaccinated animals. Some differences were noted in the complement and inflammasome responses post-vaccination, but only C3 mRNA levels remained after *M. bovis* infection, which was associated with reduced pathology (**Figure 3B**) (37).

##### 3.2.2.4 Invertebrates

In recent years, invertebrate animal models such as the insect *Galleria mellonella* (99, 118, 119) and planarians (*Dugesia japonica* and *Schmidtea mediterranea*) (91) have been used to study TB. These smaller models are advantageous for high-throughput evaluation of innate immune responses and align with animal research’s 3Rs principles (Replacement, Reduction, and Refinement) (120). Invertebrates, which lack a well-developed adaptive immune system, rely primarily on robust innate immunity to defend against pathogens and other stressors, making them valuable for studying innate immune responses against bacteria, including MTBC (121). Insect models, for example, have contributed significantly to understanding innate immunity and facilitated the discovery of mediators, such as the Toll-like receptors (TLRs). Conserved immune responses across insects and mammals include phagocytosis, opsonization, oxidative stressors, and eicosanoid synthesis, among others yet to be characterized (121, 122).

###### 3.2.2.4.1 Galleria mellonella

The larvae of *Galleria mellonella* (greater wax moth) were used to assess infection by injecting the bacteria into the hemocoel(**Figure 2A**). This model has a simple body structure and ability to tolerate human body temperature, which allows the identification of components of innate responses that are shared with more complex animals, including cells, soluble factors, cell receptors, physical barriers, and processes (like phagocytosis, cell adhesion, redox responses, and others) (123, 124). Despite the advantages, the model lacks relevance for exploring human-specific infection routes and the interplay between innate and adaptive immunity. However, the lack of adaptive responses and the relatively short time as a larvae could be exploited to study “pure” early innate or “sustained” responses (up to 6 weeks approximately, when the next pupa stage start to develop) (125).

Pathologically, *G. mellonella* larvae exhibit granuloma-like structures (called nodules) in their fat body with infected hemocytes (insect immune cells) (122). Hemocytes are phagocytic cells (similar to human macrophages and neutrophils) and were described in two out of three articles reporting infecting insects (118, 119). Different subtypes of hemocytes have been described (i.e., granulocytes, spherulocytes, plasmatocytes, prohemocytes, coagulocytes, and oenocytoids) (126, 127) with the identification of distinct cell markers (128, 129). However, the specific roles of different hemocyte subtypes in the immune response against MTBC remain unclear (118). Additionally, this model has potential for studying “trained immunity,” as BCG vaccination improves survival in larvae, hinting at its relevance for exploring innate induced memory in TB (99).

###### 3.2.2.4.2 Planarian species

Planarian species, specifically *D. japonica* and *S. mediterranea*, were explored as a novel model for studying *Mtb* infection. These planarians were infected directly by exposing them to liver homogenates mixed with *Mtb*. However, the study found that planarians could resist *Mtb* infection, as no viable bacteria were recovered after nine days post-infection (91). A fundamental limitation of this model is that temperatures above 30°C are lethal for these planarians (130), which does not match the optimal growth temperature of *Mtb* (37°C). This temperature difference may have impacted the growth and survival of *Mtb* during infection. Specific findings regarding this model were focused on autophagy, which will be discussed in section 3.2.3 under the subsection “*Autophagy-related proteins*.”

#### 3.2.3 Soluble and membrane-associated factors distributed in innate pathways

This section aims to provide information about the different factors (soluble and membrane-associated) studied by classifying them in different immunological pathways. Some pathways contain mediators acting on both innate and adaptive responses, like IL-21 or IFN-γ. However, only specific findings in the evaluation of innate responses were selected. For instance, IL-21 was evaluated as part of γδ T-cell response (46).

##### 3.2.3.1 Tumoral necrosis factor alpha (TNF-α) pathway

One of the central and most frequently identified factors in these reviewed articles was the cytokine TNF-α that was reported in articles using mice (40, 43, 48, 50, 71, 72, 75–83, 131), NHP (73), and rabbits (74). This pathway is responsible for multiple biological functions, including inflammation, cell proliferation, differentiation, and apoptosis (132). TNF-α is a cytokine produced by immune and non-immune cells and has a crucial role in TB protection (9). Signaling involves different molecules, such as the transcriptional factor Nf-kB. Nf-kB was increased in animals treated with sphingolipid sphingosine-1-phosphate (S1-P) that exhibited protection to *Mtb* (84). Most of the trends reported for TNF-α, were similarly reported for IFN- γ, except for one study in which the Mif-/- animals were used (**Table 1**). Significant trends identified in this pathway are described in the first section of **Table 2**, while those obtained in KO mice strains and NHP are summarized in **Tables 1** and **3**, respectively.

##### 3.2.3.2 ROS and RNS production in phagocytes

Reactive oxygen species (ROS) and nitrogen species (RNS) are essential components of the antimicrobial system, produced mainly by macrophages (9, 133). iNOs was one of the most frequently reported proteins in this category. iNOs was higher in ATB in different mammals evaluated (**Table 2**). Different articles described the role of iNOs during anti-mycobacterial defense, being stimulated by type-I IFN (86), S1-P (84), and cytokine-inducible SRC homology 2 (SH2) domain protein (CISH) (40). Conversely, overexpression of scaffold/matrix attachment region binding protein 1 (SMAR1) induced a significant reduction of iNOS expression in the lungs of infected mice at six w.p.i (87). In the same article, increased levels of IFN-γ and IL-12 in lung homogenates at four w.p.i were also reported, suggesting responses associated with adaptive immunity and potentially “sustained” innate immunity. These findings highlight the need to not only evaluate the kinetics of release, but also identify the cell where those innate responses are originating *in vivo.*

Neutrophil cytosolic factor 4 (NCF4) is a component of the NADPH oxidase complex, which is predicted to be involved in the production of O_2_^-^, via NADPH oxidase 2 (134). Increased NCF4 levels were related to reduced bacterial load and a protective response in animals immunized with an *Ipr1*-modified BCG vaccine (a BCG carrying the mouse gene *Ipr1-*intracellular pathogen resistance 1) (88). Other proteins and their trends are also summarized in **Table 2**.

Lastly, the production of ROS by lung-residing myeloid cells (primarily PMN and AM) in mice was hampered by the early action of platelets that reduced the availability of these cells by forming aggregates in the lung by 21 d.p.i (independently of their canonical activation, by cyclooxygenase (COX)-1, glycoprotein IIb/IIIa or the ADP-receptor P2Y_12_). This effect was confirmed by reduced bacterial load and lung pathology, as well as increased survival following platelet depletion around the onset of inflammation at seven d.p.i, but not before the infection (135). Increased early platelet response has been also reported in human patients that progress to ATB (136).

##### 3.2.3.3 IFN signaling

There are three types of IFNs: Type I (mainly IFN-α, -β, and others), Type II (IFN-γ), and Type III (IFN-λ). Reduced levels of IFN-γ were reported in diabetic mice as early as 24 h.p.i, that were exhibiting a dysregulated immune response with increased bacterial burden and inflammatory lesions (**Figure 3A**) (71, 72). In NHP, type I and type II IFN transcriptomic signatures were associated with ATB as early as two w.p.i (**Table 3**) (104). Additional molecules and associated articles are summarized in **Table 2**. Also, the interaction between this pathway and the neutrophil response was studied in the deficient IFNγR−/− mice (**Table 1**).

IFN regulatory factors (IRF 1, 2, 3, 4, 5, and 7) showed a diverse response during *Mtb* infection. IRF7, among other innate transcriptional signatures from PMB cells in the blood, were correlated with the extent of disease and were strongly reduced in the vaccinated RM that exhibited *Mtb* control and reduced pathology (**Table 3**) (93). In the article by Javed et al., IRF1 was highlighted among the early blood transcriptomic biomarkers in active TB that was also shared between human and macaque pathways as part of the type II IFN signaling, with sustained upregulation from the second through the 12th-w.p.i. Other IRFs, such as IRF2 and 4, were expressed differently, depending on the time point evaluated and the lineage of the infected animal (either Chinese or Mauritian macaque lines) (**Table 3**) (104).

##### 3.2.3.4 Neutrophil recruitment

Neutrophils can internalize bacteria and activate inflammatory and effector responses, by the action of mediators like myeloperoxidase, elastase, and the production of NETs. As it was discussed earlier, neutrophils generate one of the earliest responses in the guinea pig (**Figure 3**, **Table 2**); however, they were ineffective at eliminating *Mtb* (90). Other publications concluded that the role of neutrophils is not necessarily bactericidal but perhaps in initiating inflammatory responses (29). Das et al. and Moreira-Texeira et al. concluded that neutrophil accumulation without a macrophage response leads to higher bacterial burden, increased disease severity, and greater lethality. This macrophage-reduced response was observed in MIF deficient mice (simultaneous to reduced macrophage innate cytokines: IL-6 TNF-α, IL-12, IL-10) (50, 54).

Other proteins also associated with this pathway are described in **Table 2**. Among those, proteolytic matrix metalloproteinases (MMP) participate in the degradation of the lung extracellular matrix components, with the subsequent *Mtb* spread. These proteins have been proposed as biomarkers of ATB disease (137). During our review, we found enhanced MMP-1, 8, and 9 levels in blood and lungs during active TB that was responsible for excessive inflammation and tissue damage in mouse-susceptible strains (54). MMPs were also highly expressed during neutrophil activation and recruitment and induced neutrophil responses such as the NETs (43, 54, 85).

The dual role of neutrophil described in the reviewed articles is represented by excessive inflammation in acute phase after infection (probably bacterial dose/strain-dependent) (56) and protection in “sustained” or trained immunity after BCG vaccination in mice (29). In humans, neutrophil degranulation and NETs markers are also increased early in patients that progress to ATB (136). In **RM**, a higher expression of neutrophil degranulation makers was seen before exposure to *Mtb* that resulted in reduced lung pathology and bacterial load after infection, and therefore categorized as a protection marker (pre-infection) (**Table 3**) (93). This protective role of neutrophils may be driven by a specific subpopulation that could be induced since early developmental stages in the bone marrow after BCG immunization (29, 35).

##### 3.2.3.5 Inflammasomes

**Figure 4:**
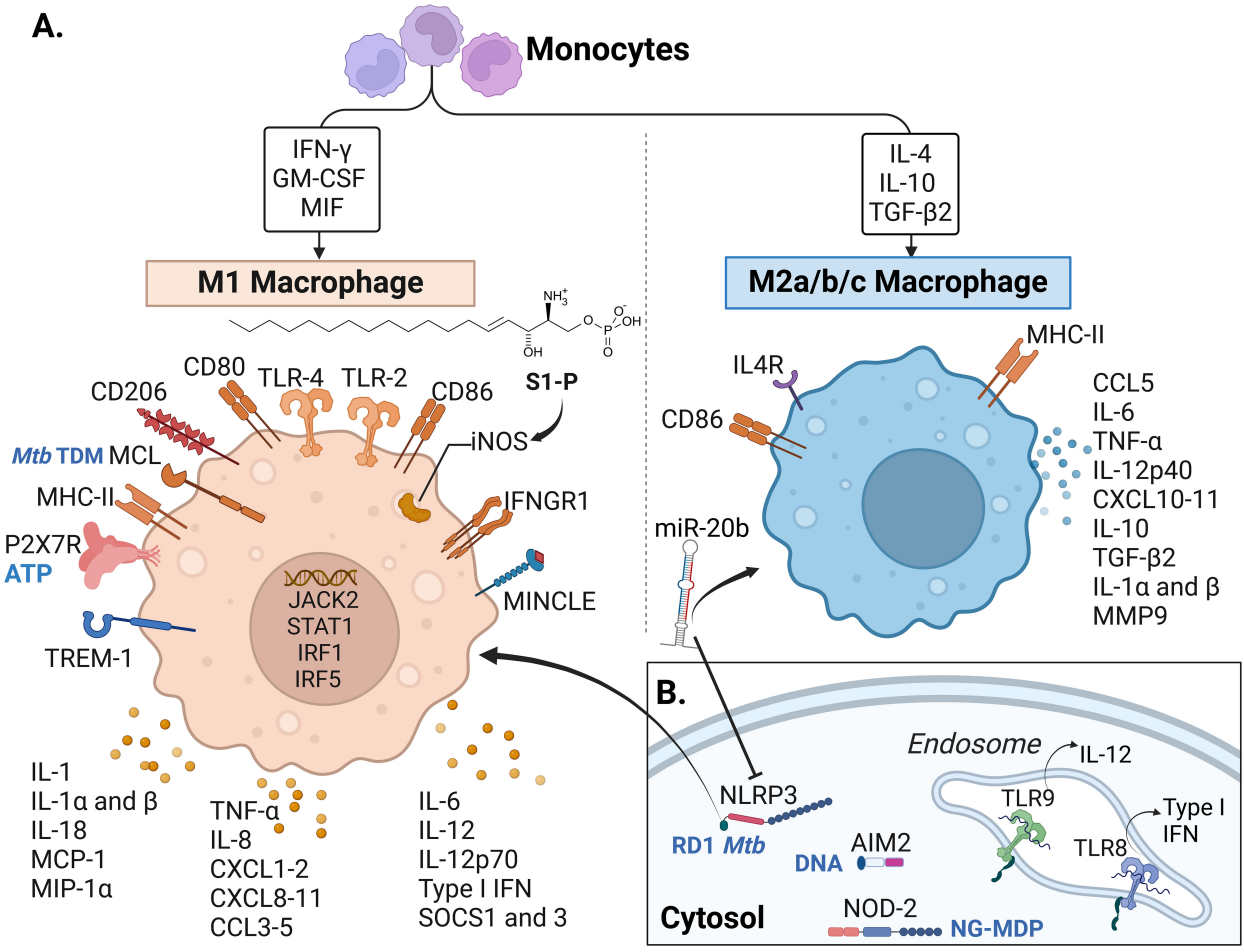
Macrophage receptors and soluble factors identified in response to *Mtb* complex in the reviewed articles.A. Cytokines, chemokines and receptors identified in the reviewed articles mostly associated with M1 macrophages. **(B)** Intracellular and endosomal receptors. Some of the recognized molecules (from *Mtb* or the host) are written in blue in panels **(A, B)**, including TDM: trehalose dimycocerosate (*Mtb*), ATP: Adenosine triphosphate released from host damaged cells, RD-1 *Mtb*: region of difference 1, present in virulent *Mtb* strains, DNA: self (host) and bacterial DNA, NG-MDP: Mycobacterial N-glycolylated muramyl dipeptide. IFN, Interferon; GM-CSF, Granulocyte-monocyte colony-stimulating factor; MIF, Macrophage migration inhibitory factor; IL, interleukin; TGF, Transforming Growth Factor; S1-P, sphingolipid sphingosine-1-phosphate; TLR, Toll-like receptor; iNOS, inducible nitric oxide synthase; IFNGR, Interferon-gamma receptor; MINCLE, Macrophage inducible C-type lectin, also known as CLEC4E; TREM, Triggering receptor expressed on myeloid cells; P2X7R, P2X purinoceptor 7; MHC, Major histocompatibility complex; MCL, macrophage C-type lectin; MCP-1, monocyte chemotactic protein-1; MIP-1α, Macrophage inflammatory protein-1α; CXCL, chemokine (C-X-C motif) ligand; CCL, C-C chemokine ligand; SOCS, Suppressor of cytokine signaling; MMP, Matrix metalloproteinase; miR-20b, microRNA 20b; NLRP3, NOD-like receptor (NLR) pyrin domain-containing protein 3; AIM2, DNA cytosolic sensor “absent in melanoma 2”; NOD, Nucleotide oligomerization domain. Macrophage phenotype classification done following (140–143). Created with BioRender.com.

The inflammasome is a complex consisting of a sensor (such as a cytosolic pattern recognition receptor or PRR), an adaptor, and an effector (pro-caspase-1) proteins that, when activated, cleaves pro-inflammatory cytokines essential for MTBC control (138, 139). We found articles describing two types of inflammasome receptors: “NOD-like” receptor (NLR) pyrindomain-containing protein 3 (NLRP3) and the DNA cytosolic sensor “absent in melanoma 2” (AIM2) (**Table 2**). In mice, NLRP3 interacts with the *Mtb* Esat-6 protein and influences the expression of IL-18 and IFN-γ (94). In addition to mice (94, 95), the inflammasome complex NLRP3/caspase-1/IL-1β was reported in wild boars (37). In that model, mRNA levels of these inflammasome-associated molecules were increased in the PBMC from oral mucosa after oral vaccination with BCG (**Figure 3B**) (37). On the other hand, NLRP3 was shown to be mitigated by miR-20b binding to the 3’-UTR of NLRP3 mRNA, inducing M2 macrophage polarization in the mouse (**Figure 4**) (95).

Another inflammasome-related PRR is the AIM2-like receptor. Kuptz et al. demonstrated that NLRP3 rather than AIM2 was involved in the activation of IL-18 in mice (94). In NHP (**RM**), AIM2 was increased in animals with active TB and reduced in vaccinated animals that showed *Mtb* control (no signs of granulomatous disease) (**Table 3**) (93).

##### 3.2.3.6 Autophagy

More than 30 autophagy-related genes (ATGs) and other players, such as the microtubule-associated protein 1A/1B-light chain 3 (LC3), perform this innate protein degradation and regulatory mechanism in mammals. The activation of these proteins results in the membrane invagination of the bacteria, its products, or even damaged self-organelles. There is also a non-canonical autophagy known as LC3-associated phagocytosis (LAP), with the participation of PRR, independently of autophagosome formation (144). Deficient ATG5 mice exhibit more severe inflammation and succumb earlier to the infection (**Table 1**) (53). More recently, Kinsella et al. reported that ATG5 was required in CD11c^+^ cells (lung macrophages and DCs) to control the production of pro-inflammatory cytokines and chemokines and avoid neutrophil recruitment (81).

LC3 participates in the autophagosome maturation and has two recognized isoforms: a cytosolic (LC-I) and a membrane-associated isoform (LC3-II, that is LC-3 conjugated to phosphatidylethanolamine) (145). LC3-II, regularly measured as marker of autophagic activity, was found increased in *M. bovis* infected mice after treatment with nilotinib (also discussed in section 3.2.5) (92). LC3 was evaluated also in planarians. LC3 co-localization with *Mtb* in mature phagolysosomes and LAP was fundamental for *Mtb* elimination in planarians. Finally, the Membrane Occupation and Recognition Nexus repeat-containing-2 (MORN2) transcript promoted both LC3 processes, and *Mtb* phagocytosis in planarians and humans (91).

##### 3.2.3.7 Antimicrobial peptides

Increased antimicrobial peptides were reported in the early response to MTBC infection, which was more prominent when a hypervirulent strain infected the host (96, 99). Cecropins increased 48 hours post BCG infection in the insect model (99), while hypervirulent bacteria induced higher cathelicidin-related antimicrobial peptide (Cramp) and defensins response in the mouse model as soon as one d.p.i compared to the levels induced by H37Rv (**Figure 3A**) (96). In other cases, the antimicrobial activity of these peptides was evaluated in the mouse model as a potential therapeutic alternative (79, 97). For instance, Ramos-Espinosa et al. evaluated the use of recombinant adenovirus as a delivery strategy for antimicrobial peptides [Human β-defensin 3 (HβD3) or cathelicidin (LL37)]. This treatment strategy resulted in reduced bacterial load and pneumonia with higher expression of proinflammatory cytokines and a synergistic effect with anti-TB drugs (97). In line with this, KO mice for Cramp (LL37 mouse homolog) were unable to control *Mtb* and succumbed earlier after infection (44) (**Table 1**).

In the rabbit, the antimicrobial peptide HAMP was studied, which is also known to regulate systemic iron. In this study, HAMP levels in the lung were upregulated after 8 weeks post*-Mtb* infection in animals supplemented with iron without affecting bacterial load (74). Although antimicrobial peptides are mainly produced by epithelial cells and macrophages (96), none of the reviewed articles evaluated the epithelial cells *in vivo* during an MTBC infection. Lastly, lower expression of the gene *RegIII* γ, involved in defense peptide production in the intestine, was found in mice with antibiotic induced-intestinal dysbiosis. This trend was more intense after the mice were infected with H37Rv and in the infected animals that were treated with Isoniazid (INH) (**Table 2**) (76).

##### 3.2.3.8 C-type lectin receptors

These groups of PRR were represented in the reviewed articles mainly by the Macrophage inducible C-type lectin receptor (MINCLE) and macrophage C-type lectin (MCL). Macrophages and DCs express MCL that recognize *Mtb* TDM (Trehalose 6,6’-dimycolate). Reduced MCL levels were linked to increased inflammation, bacterial burdens, and mortality in the mouse model (**Table 1**) (41). These receptors exert non-redundant anti-*Mtb* function, facilitating the uptake of the bacteria and the signaling towards proinflammatory responses (41, 131). Gut microbiota dysbiosis reduces the expression of MINCLE in DCs that induces adaptive T cell CD4^+^ responses, demonstrating a strong relationship between gut microbiota and DCs with subsequent *Mtb* elimination (146). Gut microbiota disruption also reduces the expression of other innate receptors, such as NOD2, MHC-II, and TLR2 in pulmonary DCs that participate in anti-*Mtb* responses in the mouse model (76, 100, 146).

##### 3.2.3.9 Toll like receptor

We found three TLRs in our reviewed articles: TLR2, 4, and 9. Significant findings related to these TLRs and other molecules participating in this innate pathway are described in **Table 2**. The activation of TLR4 can be dependent or independent of myeloid differentiation primary-response protein 88 (MyD88). MyD88 is fundamental to clear MTBC infection. The reactivation of MyD88 signaling in myeloid cells (macrophages and DCs) during *M. bovis* BCG infection is sufficient to control pathogen growth and reinstate local inflammatory cytokine production (IL-12p40, IFN-γ, and IL-1β in lungs) in mice (103). Before *Mtb* infection, Myd88 and the Toll/IL-1R domain-containing adaptor-inducing IFN-β (TRIF) transcripts were higher in vaccinated wild boars than in unvaccinated wild boars, as a sign of activation of the innate response and reduced pathology (**Figure 3B**) (37). Another mediator in TLR4-mediated responses is the tumor necrosis factor receptor-associated factor 6 (TRAF6). TRAF6 was associated with TB signs and tissue damage in the TCRβ deficient mice (**Table 1**) (43).

##### 3.2.3.10 Fc gamma receptors (FCGRs)-dependent phagocytosis

This phagocytic receptor binds to the Fc portion of immunoglobulin G (IgG). One article evaluated the neonatal Fc receptor (FcRn) in a KO mutant mouse strain (**Table 1**). *Mtb-*infected mice lacking FcRn had a reduced neutrophil infiltration in *Mtb-*infected lungs, concomitant to reduced bacterial burden and pathology. Because of the absence of FcRn reduced the capacity of CD103^+^ DCs to eliminate bacteria, other phagocytic cells may be driving the *Mtb* elimination and contributing to the low pathology profile observed (49).

##### 3.2.3.11 Immunoregulatory interactions between lymphoid and non-lymphoid cells

In this pathway, we have grouped the different IL observed in four mammalian species (**Table 4**) and the triggering receptors expressed on myeloid cells (TREM)-1. A high frequency of articles reporting IL and other cytokines and chemokines could be explained by their relevance in the inflammatory responses generated after MTBC infection, the availability of analytical methods (ELISA, Luminex^®^, flow cytometry), and reagents that allow the multiplexed study of these molecules, particularly in the mouse model. In the remaining animal models, IL and other cytokines were primarily studied using gene expression analysis, either by using qRT-PCR (40, 87), whole transcriptomic studies, or a combination of both (55, 104). The increase of acute-phase cytokines such as IL-1β, IL-6, or anti-inflammatory IL-10 in serum or different tissues of mammals depended mainly on the specific factor being studied (KO mouse strains, ATB vs LTBI, etc.) and how those induce early or “sustained” changes in innate responses (**Tables 1**, **3**; **Figure 3**, section 3.2.2). Some of the articles included in this review evaluated these innate cytokines in response to different factors, such as the use of hypervirulent strains (55, 147). For instance, IL-17 is required for early protective immunity against the hypervirulent *Mtb* HN878, but not much against *Mtb* H37Rv or CDC1551 (147).

**Table 4:** Reported interleukins (IL) associated with innate responses against *Mycobacterium tuberculosis* complex (MTBC) differentiated by animal models.

	Mouse	Non-Human Primates	Rabbit	Wild boar	Cattle
IL-1β	(42, 71, 72, 78, 103, 147, 148)			(37)	(31)
IL-6	(43, 48, 50, 71, 72, 78, 80, 82, 149–151)		(74)	(37)	(31)
IL-10	(43, 45, 50, 75, 80, 86)	(85)	(74)		
IL-12	(43, 50, 87, 102)				
IL-17A	(46, 77, 151, 152)				
IL-17	(32, 54, 82, 147)	(73)			
IL-2	(71, 72, 106)				
IL-12p40	(75, 86, 103)				
IL-4	(43, 71, 72)				
IL-1α	(86)		(55)		
IL-18	(94)		(55)		
IL-8		(104)			
IL-12b	(148)				
IL-12p70	(42)				
IL-21	(46)				

Besides myeloid cells, TREM-1 is highly expressed in Vδ2 T cells of human patients with ATB and is involved in inflammation (153). In our review, increased TREM-1 signature-associated transcripts were associated with higher inflammation and disease severity in NHP at the time of diagnosis (90-180 d.p.i), similarly observed in human studies (111).

##### 3.2.3.12 Eicosanoids and enzymes that participate in their synthesis

Eicosanoids are lipid mediators derived from arachidonic acid, produced by cyclooxygenases (COXs) and lipoxygenases (LOs), among other enzymes. These mediators include prostaglandins, resolvins, lipoxins, and leukotrienes (154–159). Eicosanoids have protective functions in innate and adaptive responses after *Mtb* infection (9, 155, 160). The most frequently reported eicosanoid, prostaglandin E2 (PGE2), generated by COX1, limits tissue damage and bacteria growth in the mouse lungs at 15 d.p.i (105). Mayer-Barber et al. similarly showed that PGE2 induced anti-*Mtb* activity and was stimulated by IL-1 during infection in mice, resulting in reduced CFU in the lung four w.p.i (86). During TB progression in susceptible mice, increased serum levels of leukotriene B4 (LTB4) and PGE2, as well as decreased levels of lipoxin A4 (LXA4) were seen in the first 30 d.p.i. LTB4 increases susceptibility to *Mtb*. Therefore, a balanced PGE2/LTB4 response influences the severity of *Mtb* infection (82). The other eicosanoid-producing enzyme, 5-LO, generates LTB4 and LXA4. In mice, 5-LO is negatively associated with protection against *Mtb*, since its absence led to higher levels of PGE2, low bacterial counts, and rearrangement of the profile of cells recruited to the lung (favoring CD11c^+^, CD19^+^, and CD3^+^/CD4^+^ cells) after *Mtb* infection (105).

#### 3.2.4 Proteins exclusively present in insects

Despite being mentioned only by Asai et al., several insect proteins were described in a discovery-mass spectrometry-based approach (**Table 5**) (99). Proteomic analyses in *G. mellonella* infected with BCG strain identified proteins like serpins (serine protease inhibitors), which play a role in innate immune responses in both insects and mammals. This and other major identified proteins with potential or known homologues in mammals are described in **Table 5**. In insects, serpins are associated with the regulation of the TLR pathway and other unique immune processes like melanization (169). Although serpin sequences between insects and vertebrates are not similar, they are known to participate in defense responses in both animal species (170). Serpins in mice regulate the structure of hypoxic granulomas by inhibiting the activity of cysteine and serine proteases, which in turn will prevent excessive tissue damage and death of *Mtb*–infected macrophages (171). Serpins were elevated in response to BCG injection after 48 hours.

**Table 5:** Insect proteins found in BCG-infected *Galleria mellonella* hemolymph compared to uninfected larvae (99) and their suggested human homologous protein.

Insect protein	Trend in infected larvae	Homologous proteins or those with similar functions in humans (or mammals)
Heat shock protein (HSP)	↓ at 4 h.p.i	HSP is a conserved family of proteins (161)
Hemolin	↑ at 4 h.p.i	Not found, but C-type lectins, Immunoglobulin superfamily, or Mannose-binding lectin (MBL) are suggested (162)
Gloverin	↑ at 48 h.p.i	Not found but could be linked to defensins or LL-37 (162).
Yellow-d	↑ at 48 h.p.i	Not found.
Insect metalloproteinase inhibitor (IMPIα)	↑ at 48 h.p.i	Not found but could be linked to Tissue Inhibitors of Metalloproteinases (TIMPs) (163).
Peptidoglycan recognition protein	↑ at 48 h.p.i	PGRP is a conserved family of proteins (164).
Prophenol oxidase activating enzyme3	↑ at 48 h.p.i	Not found. However, it has immunomodulatory functions analogous to the complement system (162, 165)
Putative defence protein Hdd11	↑ at 48 h.p.i	Not found. However, it could share functions with α- and β -defensins (162).
Cecropins D, A, C	↑ at 48 h.p.i	Defensins, LL-37, Dermcidin (162)
Serpin-2, 3a, 4B, and 11.	↑ at 48 h.p.i	Serpins are a conserved family of proteins (166)
Beta-1,3-glucan-binding protein (BGBP)	↑ at 48 h.p.i	β-1,3 glucan (162)
27KDa hemolymph	↓ at 48 h.p.i	Not found.
Lysozyme-like protein 1	↓ at 48 h.p.i	Lysozyme (LYZ) is a conserved protein (162)
Scolexin	↓ at 48 h.p.i	Not found. However, functional analogues could be C-type lectins and serine proteases (167).
Apolipophorins 1 and 2 (encoded by the same gene)	↓ at 168 h.p.i	Apolipoprotein B (apoB) (168)

h.p.i, hours post infection.Symbol ↑ stand for increased and for symbol ↓ stand for decreased.

Additionally, the protein scolexin, involved in coagulation, was reduced at the same time point (99), a contrasting trend reported in response to different infections in other insect models (167). Scolexin has shown lectin properties in other insects, which could be interesting to evaluate in the context of MTBC infection. These lectin properties are relevant due to the known interaction between the mycobacterial mannose residues and C-type lectin receptors in mammals that facilitate bacterial adhesion to cells, uptake, and intracellular persistence (172). Some of the proteins reported after BCG infection, such as the putative defense protein Hdd11, were similarly altered in invasive aspergillosis in the *G. mellonella* infection model. Hdd11 shares domains with an insect protein involved in nodulation, a process similar to granuloma formation in mammals (173). Interestingly, a similar reduction was observed for the 27 kDa hemolymph protein and Lysozyme–like protein 1 after BCG and *Aspergillus fumigatus* infection in this insect. However, the changes were 24 hours earlier in the fungal infection (173).

#### 3.2.5 Other relevant molecules

##### 3.2.5.1 Metabolites

This review explores the role of various metabolites that influence the early immune response to MTBC infection. Vitamin B5 has shown promise by reducing bacterial load, promoting macrophage maturation, and increasing pro-inflammatory cytokine levels (TNF-α, IFN-γ, IL-17) in mice as early as one-week postinfection (83). In contrast, vitamin B6 did not appear to affect the innate immune response in mice (149), and iron supplementation did not significantly alter bacterial burden or disease progression in rabbits (74). Additionally, S1-P induced M1 macrophage polarization, iNOS expression in the lungs and protection against infection (84). This review summarizes other factors influencing M1 and M2 macrophage polarization, shown in **Figure 4**. Recent findings have demonstrated that the classification into M1 and M2 macrophages is rather simplistic in explaining the events happening during infection *in vivo*; however, it is still helpful to describe an inflammatory or anti-inflammatory profile (174).

Another metabolite examined is 5-OP-RU ((5-(2-oxopropylideneamino)-6-D-ribitylaminouracil), an intermediate in bacterial riboflavin biosynthesis, which has demonstrated contrasting effects *in vitro* and *in vivo. In vitro*, 5-OP-RU activates MAIT cells, but *in vivo*, it requires co-stimulation with the TLR2/6 agonist Pam2Cys to promote pulmonary MAIT cell expansion in mice (89). In NHP infected with *Mtb*, treatment with 5-OP-RU activated MAIT cells, but did not expand them, nor did it help control the infection. In some cases, animals treated with 5-OP-RU developed acute respiratory symptoms, prompting the authors to reconsider its therapeutic potential (175). Effects of metabolites (including eicosanoids) in innate immunity should be evaluated as a sole factor, but also in combination with others (metabolites and proteins), due to the reactive nature of these molecules, their half-life and their co-stimulatory and antagonistic effect observed with other molecules. For example, the co-stimulatory activity described between 5-OP-RU and Pam2Cys (89) and the antagonistic effect observed for PGE2 and LTB4 (82). Moreover, while 5-OP-RU showed minimal impact on the intestinal microbiota in NHPs (175), its effects on the respiratory tract microbiota remain an area for further investigation.

##### 3.2.5.2 Drugs as potential modulators of innate responses

We found several drug treatments with the potential for modulating the innate immune response and inhibiting *Mtb* or *M. bovis* growth, all of them evaluated in the mouse model. Biapenem and tubastatin A activated DCs and macrophages, reducing *Mtb* growth and suggesting new therapeutic avenues that stimulate the innate immune response (75, 176). Additionally, non-steroidal anti-inflammatory drugs (NSAIDs) like aspirin and ibuprofen, particularly when administered in later infection stages, reduced pro-inflammatory cytokines, alleviated lung pathology, and decreased bacterial load (80, 82).

Hussain et al. also reported that nilotinib induced both parkin and LC3-II proteins in the lung of *M. bovis* infected animals at 63 d.p.i. In a natural infection, *M. bovis* leads to the overexpression of the Abelson tyrosine kinase (Abl), that inhibits parkin, which is crucial to promote ubiquitin accumulation around the bacilli for its elimination (92). Nilotinib is a tyrosine kinase inhibitor that promotes parking activity by inhibiting Abl (177). A protective role of parkin was also described in the KO mouse model (**Table 1**). The LC3-II increased after Nilotinib treatment also suggests the induction of autophagy, an antibacterial process described in section 3.2.4 (145). Targeting these pathways with Nilotinib resulted in reduced pathology, bacterial counts in lung and spleen, and higher survival of *M. bovis-*infected animals, highlighting its potential therapeutic benefits (92, 178).

Pharmacological inhibition of COX-2, administering Celecoxib shortly before and daily after infection, significantly reduced bacterial load in the lungs of infected mice (105). These findings highlight the potential of various pharmacological approaches to combat MTBC and possibly other non-tuberculous mycobacteria, warranting further evaluation in clinical settings.

Pretreatment with anti-TB drugs like INH and pyrazinamide (but not rifampicin) disrupted the mouse microbiota, leading to an increase in the Firmicutes phylum and reduced MHC-II expression in AM at five d.p.i, which worsened lung and extrapulmonary pathology (100). Additionally, pretreatment with a broad-spectrum antibiotic cocktail exacerbated this effect, decreasing MHC-II and CD86 expression in lung myeloid dendritic cells and likely impairing antigen presentation. Disruption of gut microbiota also resulted in higher levels of pro-inflammatory cytokines (IL-6, IL-1β, IL-12) and lower levels of IL-10, further hindering the antibacterial efficacy of the subsequent INH treatment and leading to higher bacterial loads and more severe lung pathology compared to control mice (76).

## 4 Conclusions

Our review highlights a wide range of innate immune pathways activated during MTBC infections, specifically those caused by *Mtb* and *M. bovis*. Many of these pathways are highly conserved across mammals, and some, such as phagocytosis, oxidative stress responses, and antimicrobial peptide production, are also found in insects. Larvae of *G. mellonella* was proposed as a cost-effective and ethical model to study MTBC-host interactions relevant to human disease. The lack of adaptive immunity in insects (and other invertebrates), may offer valuable insights into “pure” innate immune responses. On the other hand, mice, though less susceptible to MTBC infections, are commonly used to study innate immune responses. The use of more susceptible strains and KO mouse mutants has led to discoveries of key immune mechanisms, such as trained immunity, the impact of metabolic co-morbidities (diabetes), and the early protection induced by specific soluble mediators (such as Cish, Parkin, Atg5, transcription factors like Bhlhe40, among others), eosinophils and receptors (CLECSF8).

Interestingly, the study of innate responses to MTBC also depends on the bacterial strain. For example, early protective factors against hypervirulent strains, like the Beijing strain, include IL-17 while a detrimental role was reported for the P2X7R receptor. Strains like HN878 lead to higher early recruitment of macrophages and PMN in rabbits, contributing to the formation of cavitary lesions.

The complexity of TB as a chronic disease makes it challenging to track time-dependent innate responses, especially as adaptive immune responses emerge after seven d.p.i. We must emphasize that these late “sustained” innate mediators were mainly evaluated after BCG vaccination and in the context of trained immunity. Articles explicitly describing trained immunity in mice and calves highlight the role of airway macrophages (IM and MdM) and neutrophils and the independence of NOD1 and 2 receptors and NK. The protective effect of many of the “sustained” innate responses driven by BCG vaccination in mammals and insects should be evaluated in the context of a later *in vivo* infection, in addition to *ex-vivo* experiments. Epigenetic changes associated with these memory-innate responses should be confirmed *in vivo* in different cell types using high-resolution methods to explore temporospatial histone, DNA, and RNA modifications (179).

One of the earliest innate responses observed occurs within 30 minutes of MTBC infection and involves neutrophils. Neutrophil activation emerged as one of the most frequently studied pathways, though it was often linked to excessive inflammation and disease severity. Interestingly, neutrophils were also found to have a protective role in early infection in BCG vaccinated mice. A key factor in this response was prg2 (**Table 2**), which is also involved in the eosinophil response. The protective role of eosinophils was further evaluated in mice and NHP.

Finally, recent research has expanded our understanding of the gut microbiota’s role in impairing innate immune responses towards MTBC and how the use of broad-spectrum antibiotics (pre-infection) can modulate these responses by altering gut microbiota composition. Additionally, the identification of specific innate pathways or mediators could help us to identify early or “sustained” responses associated with disease severity or protective responses. These biomarkers could be relevant in the evaluation of disease progression or immunization efficiency. Protective responses could be dynamic, as immunization could induce different responses depending on the age at which the vaccine is administered. One limitation of this review is its exclusive focus on *in vivo* studies, which excludes valuable findings from *in vitro* or organoid models. Due to the extent of this topic and the PICO question we established for the systematic review, we could not explore some of the mechanisms behind the responses measured in greater detail. Moreover, the role of sex differences in immune responses was not addressed, which represents a critical area for future research.

## Data Availability

The original contributions presented in the study are included in the article/**Supplementary Material**. Further inquiries can be directed to the corresponding authors.
